# SIMPLIFIED LAPAROSCOPIC GASTRIC BYPASS WITH GASTROJEJUNAL LINEAR MECHANICAL ANASTOMOSIS: TECHNICAL ASPECTS

**DOI:** 10.1590/0102-6720201600S10022

**Published:** 2016

**Authors:** Mariano PALERMO, Edgardo SERRA

**Affiliations:** Division of Bariatric Surgery, Centro CIEN - Diagnomed, Affiliated to the University of Buenos Aires, Buenos Aires, Argentina.

**Keywords:** Gastric bypass, Technique, Anastomosis, surgical

## Abstract

**Background:**

Gastric bypass is a restrictive and malabsorptive surgery. The restrictive part consists in the creation of a small gastric pouch. The gastrointestinal bypass serves as the malabsorptive element.

**Aim:**

To describe a simplified gastric bypass approach for morbid obese patients, showing our results, and also remarking the importance of this technique for reducing the learning curve.

**Method:**

The patient is positioned in a split legs position and carefully strapped to the operating room table, with the surgeon between the patient's legs. Five trocars are inserted after pneumoperitoneum at the umbilicus. Dissection of the esophagogastric angle and lesser curvature is mandatory before the gastric pouch manufacturing. This pouch is done with two blue load staplers. Using a blue load linear stapler inserted only half way into the hole in the pouch is used to perform the gastrojejunal anastomosis and in order to create an anastomosis that is about 2 cm in length. A side-to-side jejunojejunostomy is done with a white load linear stapler. The last step of the gastric bypass consists in the cut of the jejunum between the two anastomosis with a white load linear stapler. Blue test is performed in order to detect leaks.

**Results:**

From January 2012 to December 2015, 415 simplified RYGB were performed. Gender: 67% female and 33 % males. Average of BMI 44.7. Mean age was 42 years old. Mean operative time 79 min. 39 % of this sample had T2 diabetes. Regarding complications were observed, one fistula, one gastrojejunal stenosis and one obstruction due to a bezoar.

**Conclusion:**

The described technique is a simplified approach in which all the anastomosis are performed in the upper part of the abdomen, allowing the surgeons to be more systematized and avoiding them to make mistakes in the confection of the Roux-en-Y anastomosis. This simplified gastric bypass is a safe and reproducible technique.

## RESUMO

**Racional:**

Bypass gástrico é cirurgia restritiva e malabsortiva. A parte restritiva consiste na criação de uma pequena bolsa gástrica. O bypass gastrointestinal serve como o elemento malabsortivo.

**Objetivo:**

Descrever uma abordagem de bypass gástrico simplificado para pacientes obesos mórbidos, mostrando os resultados, e também destacando a importância desta técnica para reduzir a curva de aprendizagem.

**Método:**

O paciente é posicionado em posição de pernas abertas e cuidadosamente amarrado à mesa da sala de operação com o cirurgião entre as pernas. Cinco trocárteres são inseridos após pneumoperitônio no umbigo. Dissecção do ângulo esofagogástrico e curvatura menor é obrigatória antes da realização da bolsa gástrica. Esta bolsa é feita com dois grampeadores de carga azuis. Usando um grampeador carga linear azul inserido apenas a meio caminho para dentro do orifício na bolsa é executada a anastomose gastrojejunal a fim de criar anastomose de cerca de 2 cm de comprimento. Jejunojejunostomia laretolateral é feita com um grampeador carga linear branca. A última etapa do bypass gástrico consiste no corte do jejuno entre as duas anastomoses com um grampeador carga linear branco. Teste de azul é realizado de modo a detectar vazamentos.

**Resultados:**

De janeiro de 2012 a dezembro de 2015, foram realizadas 415 RYGB simplificadas. Gênero: 67% homens e 33% mulheres. A média do IMC foi de 44,7 e a de idade 42 anos. A média de tempo operatório foi de 79 min. 39% desta amostra tinha diabete melito tipo 2. Quanto às complicações foram observadas uma fístula, uma estenose gastrojejunal e uma obstrução devido à bezoar.

**Conclusão:**

A técnica descrita é uma abordagem simplificada, na qual todas as anastomoses são realizadas na parte superior do abdome, permitindo aos cirurgiões serem mais sistematizados e evitando cometerem erros na confecção da anastomose em Y-de-Roux. Este bypass gástrico simplificado é técnica segura e reprodutível.

## INTRODUCTION

Gastric bypass is a restrictive and malabsorptive surgery. The restrictive part consists in the creation of a small gastric pouch, which causes the sensation of satiety. The gastrointestinal bypass serves as the malabsorptive element. The length of the bypass determines the degree of macronutrient malabsorption^10^ ^,^ ^14^. In order to avoid bile reflux, the biliary limb is around 70 cm, and the alimentary limb is 120 cm. When used in metabolic surgery for the treatment of type 2 diabetes mellitus (T2DM), the extensions are 100 cm for the biliary and 150 cm for the alimentary limb. 

The aim of this study was to describe the simplified gastric bypass approach described by Almino Ramos and show our series and results, and also remarking the importance of this technique for reducing the learning curve using it routinely.

## METHOD

### Technical aspects

#### Patient and trocar position

**FIGURE 1:**
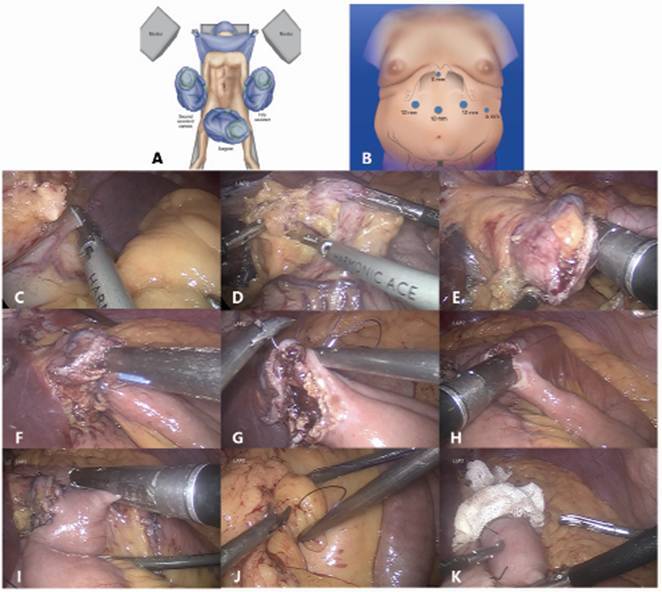
Surgical steps of simplified gastric bypass: A) patient position: B) trocar position; C) esophagogastric angle dissection with ultrasonic shears; D) lesser curve dissection with ultrasonic shears; E) gastric pouch manufacturing using blue loads; F) a 2 cm gastrojejunal anastomosis by using blue loads; G) running suture using Vicryl(r) 3.0 for the closure of the defect; H) side-to-side jejunojejunostomy performed with a white load; I) last step of the surgery, cutting the jejunum between the two anastomosis; J) closure of the Petersen defect; K) blue test is performed before the last cut of the jejunum

The patient is positioned in a split legs position and carefully strapped to the operating room table, with the surgeon between the patient's legs. The laparoscopy tower is placed on the right of the patient's head^7^ ^,^ ^12^ ^,^ ^13^ ^,^ ^14^. The surgery is performed under general anesthesia with endotracheal intubation. Abdominal insufflation up to 15 mmHg was obtained with the insertion of a Veress needle at the patient's umbilicus^6^ ^,^ ^9^. Trocars were placed as followed: 10 mm just to the left of the midline; 12 mm in the right upper quadrant on the mid clavicular line; 12 mm in the left upper quadrant on the mid clavicular line at the same level as the optical trocar; 5 mm used for liver retraction just distal and to the left of the xyphoid; and finally, 5 mm on the left anterior axillary line 5 mm distal to the costal margin (Figure 1A and 1B).

#### Dissection of the esophagogastric angle

The patient is placed in reversed Trendelenburg. The liver is retracted with a 5 mm grasper inserted through the subxyphoid port^1^ ^,^ ^8^ ^,^ ^5^ ^,^ ^14^. The first step of the surgery is to dissect the esophagogastric angle; the left diaphragmatic crus is exposed by caudal traction on the stomach fundus by the assistant to the patient's left; and the phrenogastric ligament is incised at the level of the angle (Figure 1C).

#### Dissection of the lesser curvature

After the previous step, the dissection is then continued at the lesser curvature. The second vessel is identified at this point and a surgical space is created between Latarjet's pedicle and the serosa of the stomach^2^ ^,^ ^9^ ^,^ ^11^ ^,^ ^14^. The dissection continues posteriorly and the lesser sac is entered (Figure 1D). 

#### Gastric pouch manufacturing

The stomach is transected horizontally at the level described above firing a blue load linear stapler introduced through the right upper quadrant 12 mm trocar. The stapler is introduced in the left upper quadrant 12 mm trocar and aiming the left lateral horizontal section towards the esophagogastric angle. Two stapler firings disconnect the small upper stomach from the remaining portion of the stomach. The vertical resection is performed as close as possible to the lesser curve by using a 34 Fr bougie (Figure 1E). 

#### Gastrojejunal anastomosis

The omentum is divided and the duodenojejunal angle is identified. From that site, the bowel was lifted towards the hiatus and a loop was identified that could reach this level with acceptable traction. The gastrojejunal anastomosis is about to be performed^5^. For this step, a blue load linear stapler is inserted only half way into the pouch hole in order to create an anastomosis that is about 2 cm in length before firing. This anastomosis is located on the posterior part of the gastric pouch and with 2 cm in diameter (Figure 1F). A running suture using Vicryl^(r)^ 3.0 is used to close the stapler openings (Figure 1G)

#### Measurement of 120 cm of the alimentary loop

A bowel extension of 120 cm is measured on the alimentary loop, from the gastrojejunal anastomosis. The alimentary loop at this point is secured to the biliopancreatic loop with Vicryl^(r)^ 3.0 suture.

#### Side-to-side jejunojejunostomy

Enterotomies for stapler introduction are made on the alimentary and biliopancreatic loops with ultrasonic shears. A white load linear stapler is introduced into the enterotomies and the stapler is fired. A running Vicryl^(r)^ 3.0 suture is done to close the enterotomies's opening. (Figure 1H). The last step of the gastric bypass consists in the cut of the jejunum between the two anastomosis with a white load linear stapler (Figure 1I). The advantage of this technique is that all the anastomoses are performed in the upper part of the abdomen, being more simplified and avoiding wrong anastomosis.

#### Closure of the mesenteric defect

All the mesenteric defects are closed in order to avoid internal hernias. Figure 1J shows the closure of the Petersen defect^2^.

#### Leak blue test

Blue test is done before performing the last small bowel division in order to test both anastomosis at the same time. After the blue test is done, the last stapler is fired in the jejunum (Figure 1K) ^2^ ^,^ ^3^ ^,^ ^14^.

## RESULTS

In the period from January 2012 to December 2015, we performed 415 simplified RYGB. Gender: 67% female and 33 % males. The average of BMI was 44.7 kg/m^2^. The mean age was 42 years old. The mean operative time in minutes was 79. 39 % of this sample had T2 diabetes. All the mesenteric defects were routinely closed. In this series we had no internal hernias. Regarding complications, we had one fistula reported of the gastrojejunal anastomosis, which required a re-laparoscopy. Another complication seen was a stenosis of the gastrojejunal anastomosis treated by endoscopic dilatation with only one session. The last complication seen was an obstruction due to a bezoar, treated by endoscopy.

## DISCUSSION

The Roux-en-Y gastric bypass (RYGB) is still the most worldwide performed procedure and considered the gold standard technique. The first RYGB was described in 1967 by Mason, as horizontal gastroplasty and a Billroth II gastroenterostomy. It has been undergoing a series of changes and technical improvements over time, resulting in the modern concept of vertical pouch, reconstruction in Roux-en-Y and calibrated anastomosis. The biggest change occurred with the introduction of the laparoscopic surgery, providing a signicant reduction in morbidity^4^.

Wittgrove and Clark in 1994, reported the first series of RYGB performed laparoscopically. At the beginning the gastroenterostomy was performed with a circular stapler. At that time, operations were long, about 3-4 h and technically very demanding. A new develop of the technique was described by Almino Ramos, known as "simplified RYGB" or "Brazilian technique"^4^.

The gastroenterostomy is considered the most important step of RYGB, since its size seems to be a major factor in the loss of weight of the morbidly obese patient. Studies indicate that proper size, with less than 2 cm caliber, contributes to proper weight loss, while larger than 2 cm or suffering dilation over time anastomoses may result in failure in weight loss or even weight regain. Moreover, gastroenterostomy is the most frequent site of more serious and severe complication of RYGB: digestive fistula^4^.

From the beginning the simplified RYGB, described by Almino Ramos, proved good applicability for teaching and training in bariatric surgery, even with the possibility of shortening the learning curve because all the anastomosis are supramesocolic in the upper part of the abdomen, reducing surgical time and improving outcomes. This technique was quickly adopted by several teams after Gastro Obeso Center described it, as a technique of choice. Also came to be used as a model in training courses for Brazil and other countries, getting known internationally as "Brazilian technique" for performing laparoscopic RYGB^4^.

In our group we started performing the Brazilian technique since 2011; before we used the clasic laparoscopic non-simplified RYGB. Since this date we have done 415 simplified RYGB. This technique allowed us to do a standarized technique and easier to teach to all the members of our team.

## CONCLUSION

The described technique is a simplified approach in which all the anastomosis are performed in the upper part of the abdomen, allowing the surgeons to be more systematized and avoiding them to make mistakes in the confection of the Roux-en-Y anastomosis. This simplified gastric bypass is a safe and reproducible technique and we use it routinely in our center.

## References

[1] Acquafresca P, Palermo M, Duza G, Blanco L, Serra E (2015). Gastric Bypass versus Sleeve gastrectomy: comparison between type 2 Diabetes weight loss and complications. Review of randomized control trails. Acta Gastroenterol Latinoam.

[2] Acquafresca PA, Palermo M, Rogula T, Duza GE, Serra E (2015). Early Complications After Gastric By-Pass: a literature review. Arq Bras Cir Dig.

[3] Torquati Alfonso Comparative studies an metabolic effects of sleeve gastrectomy. Duke Surgery.

[4] Ramos Almino Cardoso, Silva Andrey Carlo Sousa, Ramos Manoela Galvão, Cansaco Edwin Gonzalo Claros, Manoel dos Passos Galvao Neto, Menexes Mariano de Almeida, Galvao Thales Delmondes, Bastos Eduardo Lemos de Souza (2014). ABCD Arq Bras Cir Dig.

[5] Henry Buchwald, Estok Rhonda, Fahrbach Kyle, Banel Deirdre, Michael D. Jensen, Pories Walter, Bantle John, Sledge Isabella (2008). Weight and type 2 diabetes after bariatric surgery: systematic review and meta-analysis. The American Journal of Medicine.

[6] Cadière GB, Himpens J, Dapri G (2005). Laparoscopic stomach bypass surgery. Chirurg.

[7] Collins BJ, Miyashita T, Schweitzer M, Magnuson T, Harmon JW (2007). Gastric bypass: why Roux-en-Y? A review of experimental data. Arch Surg.

[8] Franco JV, Ruiz PA, Palermo M, Gagner M (2011). A review of studies comparing three laparoscopic procedures in bariatric surgery: sleeve gastrectomy, Roux-en-Y gastric bypass and adjustable gastric banding. Obes Surg.

[9] Himpens JM (2004). The gastrojejunostomy in laparoscopic Roux-en-Y gastric bypass. Semin Laparosc Surg.

[10] Jacob BP, Gagner M (2003). New developments in gastric bypass procedures and physiological mechanisms. Surg Technol Int.

[11] Bussetto Luca, Dixon John, Luca Maurizio De, Shikora Scott, Pories Walter, Angrisani Luigi (2014). Bariatric surgery in class I obesity: a position statement from the International Federation for de Surgery of Obesity and Metabolic Disorders. Obesity Surgery.

[12] Madan AK, Harper JL, Tichansky DS (2008). Techniques of laparoscopic gastric bypass: on-line survey of American Society for Bariatric Surgery practicing surgeons. Surg Obes Relat Dis.

[13] Palermo M, Dapri G (2015). Single Port Laparoscopic Surgery.

[14] Palermo M, Gimenez M, Gagner M (2015). Laparoscopic gastrointestinal surgery. Novel techniques, extending the limits.

